# Fingertip Injuries and Amputations: A Review of the Literature

**DOI:** 10.7759/cureus.8291

**Published:** 2020-05-26

**Authors:** Abdal Kawaiah, Mala Thakur, Stuti Garg, Sanad H Kawasmi, Abbas Hassan

**Affiliations:** 1 Orthopaedics, Xavier University School of Medicine, Oranjestad, ABW; 2 Internal Medicine, Xavier University School of Medicine, Oranjestad, ABW; 3 Plastic and Reconstructive Surgery, Northwestern University Feinberg School of Medicine, Chicago, USA; 4 Orthopaedics, University of Jordan, Amman, JOR

**Keywords:** fingertip amputation, fingertip, digital amputation, fingertip injury, hand injury

## Abstract

The fingertip is defined as the part of the digit distal to the insertion of the extensor and flexor tendons on the distal phalanx. Devastating injuries to the hand occur every year that lead fingertip amputations in thousands of people. The highest incidence rates are usually seen in children less than five years old and in adults over the age of 65. There are various presentations of injury that may end up with post-traumatic fingertip amputation, including lacerations, avulsions, and crush injuries. The fingertip is vital for sensation, as it has a high concentration of sensory receptors, and hence the restoration of sensation is the most important focus of treatment. The three main goals of treatment are the restoration of sensation and durability in the tip and assuring proper bone support to allow for nail growth. Many complications can arise after fingertip amputation, including delayed wound healing, nail deformities with poor aesthetics, hypersensitivity, residual pain, cold intolerance, scar retraction, flexion contractures, chronic ulceration, infection, and flap loss. The objective of this study is to provide an overview of the anatomy of the fingertip, the presentation of fingertip injuries and their management, and complications that might arise after surgery.

## Introduction and background

Thousands of people suffer from devastating hand injuries every year, often leading to fingertip amputations. It is estimated that as many as 45,000 finger amputations are performed in the US per year with an incidence rate of 7.5/100,000 people [1,2]. A long-term epidemiologic analysis of finger amputations in the US found the highest incidence rates to be in children less than five years old and in adults over the age of 65 years [2]. In children, injuries to the fingertips account for two-thirds of all hand injuries [3]. Furthermore, a study on occupational hand injuries found that injuries to the fingers are most commonly caused by metal items and hand tools with blades [4]. Such sharp injuries can result in lacerations, amputations, or neurovascular injuries [5].

The three main goals of treatment are the restoration of sensation and durability in the tip and assuring proper bone support to allow for nail growth. The fingertip is vital for sensation since it has a high concentration of sensory receptors; hence, restoration of sensation is the preeminent focus of the treatment [6]. Moreover, the durability of the tip is essential for finger motion as well as hand action, and, finally, the allotment of nail growth is a key factor in maintaining appearance [6]. Improper treatment of fingertip amputations may lead to defects in the appearance such as hook nail deformity, and intolerance to cold, and skin tenderness [3,7]. Deficiencies may also present in the form of stiffness and long-term functional loss. In this study, we provide an overview of the anatomy of the fingertip, the presentation of fingertip injuries and their management, and complications that might arise after surgery.

## Review

Methods

We searched PubMed, MEDLINE, Embase, and Cochrane databases for studies that met our eligibility criteria. The most recent search was performed in February 2020 using the following terms in different combinations: “fingertip”, “amputation”, “injury”, “laceration”, “avulsion”, "management", and "complications". The selection and review process was done manually in a randomized and blinded fashion by three independent reviewers. The bibliographies of the studies that met the eligibility criteria for the review were also reviewed, including papers that were not present in our initial search. Eligibility criteria included peer-reviewed studies discussing the anatomy, management, or complications of fingertip amputations in the English language. In total, 23 studies were included in the final selection.

Anatomy

The tip of the digit comprises virtually all tissue components present elsewhere in the body: skin, bone, joint, synovial membrane, ligaments, tendons, tendon sheaths, arteries, veins, lymphatic channels, nerves, and advanced nervous end organs [8]. Anatomy will be discussed in the order of repair, which is most commonly the fixation of the bone, repair of the tendons, repair of the arteries, repair of the nerves, and, finally, repair of the veins.

Distally, the finger is composed of three components: distal phalanx, the soft tissue of the nail bed, and the nail edge. Taking that into account, fingertip injuries can involve skin and pulp loss with or without exposed bone. Treatment modality will depend on the geometry of the amputated digit tip (transverse, oblique, volar, or dorsal) and its level of involvement. The oblique amputation can slope anteroposteriorly as well as laterally to involve more tissue. Being a “clean-cut” vs. a “crush” injury and the size of pulp tissue defect are other factors contributing to severity [6]. The following Landmark studies have denoted injury zone classification systems: Hirase; Allen; Ishikawa et al.; Tamai [8-11]. These systems allow for guidance and clarification when looking at treatment options [12].

The Tamai study breaks down the distal phalanx into two zones: zone 1 extends from the base of the nail to the fingertip, and zone 2 extends from the distal interphalangeal joint (DIPJ) to the base of the nail. The distal phalanx can be stabilized with the amputated tip through absorbable sutures, Kirschner wires, or an 18-gauge needle [12]. Its periosteum anchors a dense, thick fibrous layer that is present under the epidermis of the whole fingertip. Fibrous collagen bands serve as anchors and allow for the pulp to be held in place to assist in traction during grip. Volarly, the pulp consists of fibroadipose tissue that is highly vascular and surrounded by fibrous pillars [13]. The perionychium is comprised of the nail bed, nail fold, and surrounding tissue, including the eponychium, which is the proximal fold that grows over the nail. Paronychium consists of the lateral folds, and hyponychium represents the epithelium just under the free edge of the nail [14]. The nail acts as a protective plate by being composed of compact flattened keratinized squamous cells, which protect the soft tissue beneath which is the nail bed, proximally the germinal matrix, and distally the sterile matrix. The germinal matrix situated at the base of the nail bed between the nail fold to the lunula of the nail produces 80-90% of the nail plate [15]. The nail bed has a thin layer called the sterile matrix that allows firm attachment with the nail plate and permits it to slide over the nail bed despite this attachment. Nail bed and matrix are also rich in glomus bodies, whose function is fingertip thermoregulation.

Tendon insertions allow for localization of trauma and provide means for replantation indications. Fingertip amputations proximal to the insertion of the flexor digitorum superficialis (FDS) are good indications. In the case of fingertip amputations, the tendons are too far to be involved and only serve for localization. The arterial supply of the fingertip consists of multiple branches that arise from the rounded distal transverse palmar arch (DTPA); its size is 0.85-0.1 mm [16]. The arch is formed by the palmar digital arteries anastomosing with each other. The digital arteries run along the sides of the digits and dorsal to the nerves. Their terminal part is located near the DIPJ-level and anastomose distal to the flexor digitorum profundus insertion. The predominant digital artery is closer to the midline; in the index and middle digits, it is the ulnar digital artery; in the ring and small digits, it is the radial digital artery. Proximally, they have a diameter of 1-1.5 mm, which is sufficient for microsurgical anastomosis [17]. The arterial network beyond the middle of the distal phalanx is small and difficult to suture [16]. The size of these branches averages 0.58-0.1 mm [16]. The DTPA radiates longitudinally three or more vessels to supply the pulp. Centrally and palmar, the distal phalanx is one or two of the largest caliber branches that run close to the midline (0.4-0.6 mm in diameter) [17]. Proximal to the DTPA arch is the dorsal proximal matrix arch, which is completed by the branches of DTPA, turning back dorsally and anastomosing to finally supply the nail plate germinal matrix. The nail bed is supplied by branches from the distal radiating vessels that pass dorsally to anastomose at the middle and distal matrix arches [18]. Venous anatomy has more variability that has not received the attention it deserves, but its importance is denoted on replantation when venous congestion poses a risk for the procedure.

The primary draining system of the fingertip is present on the dorsum of the digit. Engaging most of the drainage is the dorsal terminal vein that is present in the midline and is approximately 1.0 mm in size at the level of the DIPJ [17]. Therefore, for any flap based on the digital artery, it is advised to utilize at least 4 mm of dorsal skin proximal to the nail plate for adequate venous anastomosis [13]. The dorsal terminal vein drains distally from the veins of the nail and finger pulp to later split into two dorsal veins proximal to the DIPJ. Searching laterally for the commissural veins provides the greatest likelihood of success at all levels of distal tip amputation, with an equal mix of vessels 0.5 mm or larger and 0.4 mm or smaller. Restoration of arterial flow can help highlight the position of suitable veins. These may be volar or dorsal. On the lateral sides, commissural veins provide the best chance of reattaching the amputated fingertip, with a mix of vessels ranging from 0.3 to 0.5 mm at the level of the nail base. Restoring arterial flow can help demonstrate the position of suitable veins [18]. Less commonly addressed is the palmar venous system due to its unpredictable location of veins at the fingertip. Digital arteries do not have venae comitantes, but there are multiple anastomosing veins accompanying each artery in the subcutaneous tissue. Rapid shunting of blood flow through a true vascular plexus at the finger pulp allows for control of digital temperature. Another contributing factor to digital temperature is by the digital nerves that send off terminal branches to form the transverse arch in the palmar subcutaneous tissue of the pulp. Those branches form a plexus that is distal to the DIPJ, which is difficult to repair surgically [17]. Finally, if the tip is not replanted after injury, the loss of that plexus means that the ability to thermoregulate is compromised [18].

Presentation and diagnosis

There are various presentations of nail bed injuries that may end up with post-traumatic fingertip amputation. Laceration, avulsion, and crush injuries are the main ones [19]. Patients suffering from nail bed laceration manifesting subungual hematomas will present with bleeding under their nail plate, which may progress to its separation from the nail bed, resulting in a significant amount of pain [20]. Nail avulsion occurs when the nail plate splits of the surrounding structures making the nail bed are exposed to the external environment [21].

The compression of the nail bed and fingertip amputation may be caused by the involvement of a sharp object during a crush injury. Diverse outcomes of these injuries may be seen at the time of the presentation. It varies from distal phalanx fracture to partial or even complete fingertip amputation [22]. The most common mechanism of injury that is seen at the time of clinical evaluation involves finger crushing of the right-hand during door closure among the pediatric age group [23].

Physical examination should be performed in order to assess the function of the injured finger. It is usually performed without anesthesia to assess the sensory and motor function of the fingertip accurately. Evaluating the distal phalanx range of motion and the possibility of distal tendon injury are the major concerns of the motor examination. A 2-point discrimination test over both sides of the fingertip or light touch sensation test is used to assess the sensory function. Capillary refill and the general color of the finger are used to assess the vascularity. Careful inspection of the injury may reveal underlying deformity caused by possible fracture or dislocation.

Moreover, the physician should inspect for the extent and location of the laceration and the presence of foreign bodies, including wood, glass, or metal. Fortunately, a digital nerve block may be indicated when examining young children or for the sake of identifying injured structures such as an avulsed nail plate, limiting the accuracy of motor and sensory assessment. Injured fingers, including the amputated ones, should be evaluated radiologically by anterior-posterior, lateral, and oblique views to rule out dislocation, fracture, or the presence of a foreign body in the distal part of the finger to confirm the diagnosis [20,22].

Management: nonoperative

Nonoperative management of fingertips is one of the treatment approaches that is employed to promote secondary healing. Nonoperative management of fingertip amputations does not entail the patient going into surgery. Instead, the case is managed using simple dressings. Patients treated using the nonoperative treatment recover quickly and do not normally experience functioning issues with their fingers.

Healing by Secondary Intention

Healing by secondary intention is the approach in which the wound is left open to heal by granulation. According to Ramirez et al., patients with no bone or tendon exposed are advised on how to dress the wound appropriately to support secondary healing [24]. Adults and children with finger amputation that have not exposed the bone or tendon and have less than 2-cm skin loss are not viable for surgery [25]. As such, the wounds are left to heal without any operation being performed as the skin cannot be pulled to cover the wound. Healing by secondary intention can be more effective than other styles of treating a finger amputation. For a finger amputation that is less than 2 cm, it is recommended that healing by secondary intention is used as it is more effective and simpler. Disfigurement and tenderness of finger amputations that affect a person's ability to work normally are some of the adverse treatment outcomes that may occur among individuals. However, healing by secondary intention has been proven to offer full recovery, helping the patients to attain normal functionality.

In children with exposed bone, the favored treatment method by secondary healing involves the usual dressing of the finger and waiting for it to heal. According to Simman and Hermans, wounds that are exposed can be managed by secondary intention. Even exposed bone and tendons generate granulation, which supports healing through secondary intention [26]. Children can recover faster than adults, and there is no need to apply complex operative means to help in the recovery process of exposed bones and tendons. Champagne et al. claim that finger amputations with exposed bone take the longest time to heal [27]. Nonetheless, they heal from the gradual formation of a granulation pad that eventually covers the exposed bone. The wound begins to contract with time, and the surrounding skin expands, resulting in a scar that envelops the amputated finger [27]. In treating exposed bones, healing by secondary intention helps to attain the functionality of the fingers and offers the best cosmetic outcome. The protruding bones are not trimmed, as these would affect the functionality of the finger. Instead, granulation is left to establish and cover the bone [27]. In children, finger amputation is treated with little or no traces of the initial injury. Hence, treatment by secondary healing in children with exposed bones is effective, and children do not require any surgical operation [26,27].

Management: operative

Primary Closure 

Severe cases of fingertip amputation warrant surgical intervention in which the operative intervention facilitates the healing of the wound. Primary closure is aimed at closing the wound and entails shortening the protruding bones to make it possible to close the wound. Wang et al. noted that fingertip revision amputation is the most used method in the treatment of fingertip amputations since it is effective in achieving positive treatment outcomes [28]. The process may entail losing part of the skin, digit length, fingernail, and may also cause function issues after treatment. Management of fingertip amputation using primary closure or revision amputation aims at helping the injured person to return to work as quickly as possible and ensures that the functionality of the finger is maintained [28]. Revision amputation leads to acceptable treatment outcomes, especially regarding sensibility. However, a shortcoming of the operative approach is that the patients treated using this approach can develop cold intolerance. Despite the individuals being treated faster and enjoying standard functionality, cold temperatures can affect their functionality. Although cold intolerance may be caused by the damaged nerve rather than the treatment procedure, it is essential that the doctors understand the effectiveness of revision amputation in order to use it to manage fingertip amputation cases.

Full Thickness Skin Grafting From Hypothenar Region

Full-thickness skin grafting from the hypothenar region allows the skin to be removed from a compatible donor part to the recipient as a form of treating fingertip amputations. As noted by Simman and Hermans, the use of Esterified Hyaluronic Acid Matrix (eHAM) treatment and grafting attained complete coverage of the bone and the eventual healing of the finger [26]. Skin grafting of the hypothenar region can be successfully employed in cases where the amputated finger lacks enough tissue for covering the finger. According to Ramsey et al., full-thickness skin grafts are used in cases where secondary healing, flap repair, and primary closure are inapplicable [29]. The approach utilizes epidermis and dermis parts of the skin to apply to the amputated fingertips and flaps. The method is easy for both the surgeon and the patient and retains the architecture of the recipient's fingertips. The treatment of the fingertips and flaps using skin grafts helps to cover areas that do not have enough skin. Although the method is mainly used as a last resort, it often results in the successful management of fingertips amputations and finger flaps.

Flap Reconstruction

Flap reconstruction is another management approach that can be used in dealing with fingertip amputations. Since the skin lacks its blood supply, full-thickness skin grafting may not be appropriate in managing extensive tissue injuries. In this case, the wound is managed using the full thickness of the skin taken from a different part of the injured hand or distal part of the body [30]. Injuries with exposed bone and a lack of soft tissue for coverage often require flap reconstruction if the patient does not wish to have a complete amputation. Although it is important to preserve the finger length, reconstruction and replantation of the flaps often require a prolonged period of immobilization and recovery [13]. When a small coverage area is required, local flaps offer the lowest morbidity at the donor site. On the injured digit, local flaps are taken from a donor site comprising healthy tissue adjacent to the wound. Examples of local flaps include the Kutler lateral V-Y flap, the Atasoy-Kleinert V-Y flap, and the Moberg flap (thenar advance) [13,30].

Regional flaps use tissue not adjacent to the defect. Such flaps are useful if the wound is too large to enable coverage with a local flap. Commonly used regional flaps to manage fingertip amputations include the thenar flap, cross-finger flap, and the thenar-H flap [13]. Finally, island flaps with a neurovascular pedicle could also be used for covering the fingertips. Such flaps have two advantages that can avoid the prolonged immobilization time necessary for a cross-finger or thenar flap, and they allow the restoration of fingertip sensation [13].

Complications

Many complications can arise after fingertip amputation, including delayed wound healing, nail deformities with poor aesthetics, hypersensitivity, residual pain, cold intolerance, scar retraction, flexion contractures, chronic ulceration, infection, and flap loss [31].

Delayed wound healing is caused by inappropriate debridement of devitalized tissues and, more commonly, closure with excessive tension. This problem can occur at the wound margins following necrosis of the tissue. Secondary intention healing occurs over time from the contraction of wound and reepithelialization. Wound contraction leads to a scar that is significantly smaller than the original defect and reepithelialization with defective scar results in almost normal skin coverage [32].

Constricted scar or atrophic change on the tactile pulp can lead to hypersensitive fingertips. To avoid this problem, flap coverage with a sensate flap with proper thickness for the pulp is recommended [33]. A short nail bed with insufficient bony support of the distal phalangeal bone and soft tissue deficiency is the cause of poor aesthetics and nail deformity. A loss of bone and soft tissue in the distal phalanx occurs following the amputation of the fingertip. The redundant nail bed that folds over the tip of the phalanx terminal causes a hook nail deformity [34]. This deformity leads to fingertip discomfort or even pain, which may exclude the finger from daily activities. This deformity is of functional and aesthetic concern, especially in young women and children. There are many suggested surgical procedures to treat this deformity, including the traditional antenna procedure, composite toe pulp grafting, homodigital advancement flap, free toe tissue transfer, and oblique, triangular neurovascular osteocutaneous flap [33].

## Conclusions

The tip of the digit comprises virtually all tissue components present elsewhere in the body. Injury to the fingers is most commonly caused by metal items and hand tools with blades. Such sharp injuries can result in lacerations, amputations, or neurovascular injuries. Improper treatment of fingertip amputations may lead to complications such as defects in appearance like hook nail deformity, cold intolerance, and skin tenderness. In this study, we provided an overview of the anatomy of the fingertip, the presentation of fingertip injuries and their management, and complications that might arise post-surgery.
